# Longitudinal relationships of malnutrition risk, sleep disturbance, and fear of cancer recurrence in thyroid cancer patients: a cross-lagged panel model

**DOI:** 10.3389/fnut.2026.1882241

**Published:** 2026-08-21

**Authors:** Qixin Lian, Qiang Wu, Wenjun Jia, Lei Meng, Mengqian Zhou, Chengzhi Liang, Xiao Chen, Qi Zhang

**Affiliations:** 1 The First Affiliated Hospital of Jiamusi University, Jiamusi, China; 2 Pangang Xichang Hospital, Xichang, China; 3 Department of Thyroid Surgery, The Second Affiliated Hospital of Anhui Medical University, Hefei, China; 4 Jiamusi University, Jiamusi, China

**Keywords:** cross-lagged panel model, fear of cancer recurrence, longitudinal study, malnutrition risk, papillary thyroid carcinoma, sleep disturbance

## Abstract

**Background:**

Although papillary thyroid carcinoma (PTC) is generally associated with favorable survival, postoperative recovery often involves persistent nutritional, psychological, and behavioral challenges. Malnutrition risk, fear of cancer recurrence (FCR), and sleep disturbance are all clinically relevant in thyroid cancer survivorship, yet their temporal interrelationships remain unclear. This study aimed to examine the longitudinal reciprocal relationships among malnutrition risk, FCR, and sleep disturbance in patients after PTC surgery.

**Methods:**

A prospective longitudinal study enrolled 940 patients with PTC from January 2024 to September 2025. Assessments were conducted at 3 days, 1 month, 3 months, and 6 months after surgery; 893 participants completed all four assessments. Malnutrition risk was measured using the Patient-Generated Subjective Global Assessment, FCR using the FCR-4, and sleep disturbance using the Pittsburgh Sleep Quality Index. Longitudinal measurement invariance was tested prior to structural modeling. A cross-lagged panel model was then used to evaluate autoregressive and cross-lagged associations among the three constructs across four waves.

**Results:**

Malnutrition risk gradually increased over time, whereas FCR and sleep disturbance decreased. All three variables showed significant autoregressive effects across adjacent waves. FCR was consistently prospectively associated with subsequent sleep disturbance across all intervals. Sleep disturbance was prospectively associated with subsequent FCR during the later follow-up periods only. The association between malnutrition risk and FCR was stage-dependent.

**Conclusion:**

Malnutrition risk, FCR, and sleep disturbance showed dynamic and stage-dependent longitudinal relationships during postoperative recovery in patients with PTC. These findings support repeated screening and integrated supportive care that includes nutritional monitoring, sleep management, and assessment of FCR.

## 1 Introduction

Papillary thyroid carcinoma (PTC) is the most common histological subtype of thyroid cancer and accounts for the majority of newly diagnosed thyroid malignancies (1). Although PTC is generally associated with favorable long-term survival, its increasing incidence and relatively young age at diagnosis have made survivorship care an increasingly important clinical issue. For many patients, the completion of surgery does not mark the end of disease burden, but rather the beginning of a prolonged recovery process involving physical symptoms, repeated surveillance, endocrine adjustment, and ongoing supportive care needs (2). Thyroid cancer survivors may continue to experience impaired quality of life despite the generally good prognosis of the disease (3).

Among patients recovering from PTC surgery, malnutrition risk, sleep disturbance, and fear of cancer recurrence (FCR) may represent three clinically important yet insufficiently integrated dimensions of postoperative recovery (4–6). Malnutrition is common in patients with cancer and is associated with poorer functional status, longer hospital-stay, and reduced quality of life (7, 8). In thyroid cancer care, nutritional issues may also be clinically relevant during postoperative recovery and subsequent treatment, particularly in the context of endocrine adjustment, symptom burden, and nutrition-related management (9, 10). Sleep disturbance is also prevalent after thyroid surgery and may be influenced by postoperative discomfort, hormonal changes, and persistent cognitive and emotional arousal (11). In addition, FCR has become increasingly recognized as a central survivorship concern, even among patients with cancers that have a generally favorable prognosis.

Existing evidence suggests that FCR and sleep problems are closely related in cancer survivorship, and thyroid cancer survivors may remain highly sensitive to bodily sensations, laboratory indicators, and follow-up findings during recovery. From a clinical perspective, these symptom domains may also affect appetite, fatigue regulation, daily routines, and broader self-management, making them potentially relevant to nutrition-oriented survivorship care. However, most previous studies in this area have relied on cross-sectional designs (12–14). As a result, they can identify associations among symptoms and recovery-related factors, but cannot clarify temporal ordering, including which problem emerges earlier and whether one factor is prospectively associated with subsequent changes in another, and whether these relationships vary across key postoperative transition periods (15). These limitations are particularly important in PTC, because postoperative adaptation is unlikely to be static (16). The dominant recovery challenge may shift over time, and the interrelationships among malnutrition risk, sleep disturbance, and FCR may also evolve as patients move from acute postoperative care to community-based recovery. To address these gaps, the present study adopted a prospective longitudinal design and focused on key transition points from the acute postoperative period to community recovery in patients with PTC. The 6-month follow-up period was selected to capture the early postoperative recovery phase, during which nutritional risk may fluctuate in parallel with changes in dietary intake, symptom burden, physical functioning, endocrine adjustment, and treatment optimization. Using a cross-lagged panel model, this study systematically examined prospective temporal associations and longitudinal interrelationships among malnutrition risk, sleep disturbance, and fear of cancer recurrence across four follow-up waves. By clarifying whether these factors show unidirectional or reciprocal cross-lagged associations over time, this study aimed to provide a more precise understanding of postoperative recovery and to inform integrated supportive care, including nutrition-oriented survivorship management.

## 2 Materials and methods

### 2.1 Participants and procedures

This study was conducted in accordance with the Declaration of Helsinki, and all procedures were approved by the ethics committee of the affiliated university. Prior to data collection, physicians were consulted to assess patient eligibility for participation. Clinical information, including surgical decisions, postoperative complications, lymph node metastasis status, and cytological and pathological findings, was obtained from the attending physicians. For patients who met the inclusion criteria, the study purpose was fully explained, and written informed consent was obtained.

A prospective longitudinal study was conducted among patients with PTC admitted to the Thyroid Surgery Ward of The First Affiliated Hospital of Jiamusi University. Participants were recruited using convenience sampling between January 5, 2024, and September 15, 2025. Assessments were conducted at 3 days after surgery (W1), 1 month (W2), 3 months (W3), and 6 months (W4). The final W4 assessment was completed on March 16, 2026, and the database was locked on March 16, 2026. Data collection was performed through face-to-face interviews to ensure completeness and to document any changes in patient condition. A total of 940 patients were enrolled at W1. Follow-up assessments were primarily conducted during scheduled postoperative visits. Of the baseline participants, 922 completed W2, 907 completed W3, and 893 completed W4. The screening process is shown in Supplementary Figure 1. The reasons for non-completion included inability to contact, treatment at another hospital, or deterioration in health. The primary longitudinal analyses were conducted using complete-case data from the 893 participants who completed all four assessments. Baseline characteristics were compared between participants who completed all four waves and those who did not to assess potential attrition bias.

The inclusion criteria were as follows: (1) age 18 years or older, (2) no history of drug or alcohol dependence within the past 6 months, (3) no antidepressant use within the past 6 months, (4) ability to complete the questionnaire independently or with family assistance, and (5) a confirmed diagnosis of PTC. The exclusion criteria were as follows: (1) life expectancy shorter than 6 months, (2) inability to understand or comply with the study protocol, or inability to communicate or perform self-care, (3) participation in other ongoing mood-related studies, (4) history of severe infection, malignancy, or autoimmune disease, (5) current or past psychiatric illness or use of psychiatric medication, (6) severe suicidal ideation, (7) presence of other primary tumors at the time of surgery, and (8) severe preoperative comorbidities such as uncontrolled cardiovascular disease, pulmonary disease, diabetes, or other conditions judged by clinicians to significantly affect postoperative recovery.

### 2.2 Measures

#### 2.2.1 Sociodemographic and clinical characteristics

Participants completed a self-administered questionnaire to provide sociodemographic information, including age, sex, body mass index (BMI), marital status, and educational level. BMI was calculated as body weight in kilograms divided by height in meters squared. Clinical information was obtained from the electronic medical records. The clinical variables included postoperative serum thyroid-stimulating hormone (TSH), free thyroxine (FT4), free triiodothyronine (FT3), and surgical extent. Surgical extent was categorized as lobectomy or total thyroidectomy. Postoperative TSH, FT4, and FT3 levels were measured at 6 weeks after surgery and analyzed as continuous variables. The coding of all covariates are presented in Supplementary Table 1.

#### 2.2.2 Malnutrition risk

Malnutrition risk was assessed using the Patient-Generated Subjective Global Assessment (PG-SGA) (17, 18). The PG-SGA comprises a patient-completed component and a clinician-rated component. The patient-generated section includes weight history, food intake, nutrition impact symptoms, and activities and function. Unlike assessments based solely on body weight or BMI, the PG-SGA integrates recent weight history, changes in food intake, nutrition-impact symptoms, activity and functional status, metabolic demand, and physical examination findings, including loss of subcutaneous fat, muscle wasting, and fluid accumulation. In this study, the PG-SGA was used to identify malnutrition risk and nutrition-related symptom burden rather than to establish an etiologically specific diagnosis of malnutrition. For the food intake item, the highest applicable score was assigned, whereas symptom scores were summed. The clinician-rated section evaluates disease status in relation to nutritional requirements, metabolic stress, and physical examination findings. Scores for disease status and metabolic stress were summed, and the physical examination score was derived from the assessment of fat loss, muscle wasting, and fluid status, with the muscle loss score used as the final score for the physical examination item. The total PG-SGA score was calculated by summing the scores of all components, and a score greater than 3 was considered indicative of nutritional risk. In the present study, the Cronbach’s alpha coefficient for the PG-SGA was 0.872.

#### 2.2.3 Fear of cancer recurrence

Fear of cancer recurrence was measured using the Fear of Cancer Recurrence Scale (FCR-4). This scale is a short form derived from the original FCR-7 developed by Simard and Savard in 2018 and is designed to rapidly assess core concerns, anxiety, and intrusive thoughts related to cancer recurrence (19). The scale consists of four items rated on a five-point Likert scale ranging from “never” to “always.” Total scores range from 0 to 16, with higher scores indicating greater fear of recurrence. The Cronbach’s alpha coefficient in this study was 0.883.

#### 2.2.4 Sleep disturbance

Sleep quality was assessed using the Pittsburgh Sleep Quality Index (PSQI) (20). The validated Chinese version has demonstrated good reliability and validity (21). The PSQI consists of 23 items covering seven components: sleep duration, sleep efficiency, sleep latency, sleep disturbances, subjective sleep quality, use of sleep medications, and daytime dysfunction. The total score ranges from 0 to 21, with higher scores indicating poorer sleep quality and more severe sleep disturbances. In this study, the Cronbach’s alpha coefficient was 0.853.

### 2.3 Statistical analysis

All statistical analyses were conducted using SPSS 26.0 and AMOS 24.0. A two-tailed *p*-value < 0.05 was considered statistically significant. Prior to structural modeling, measurement invariance across time was evaluated using confirmatory factor analysis (CFA) to ensure that the latent constructs were comparable across waves (22). Configural, metric, and scalar invariance were tested sequentially by imposing equality constraints on factor loadings and item intercepts. Changes in model fit indices were used to determine invariance, with ΔCFI < 0.01 and ΔRMSEA < 0.015 indicating acceptable invariance (23).

Based on their potential associations with malnutrition risk, FCR, and sleep disturbance, age, sex, BMI, marital status, educational level, postoperative TSH, FT4, FT3, and surgical extent were included as covariates in the adjusted cross-lagged panel model. All covariates were allowed to correlate with one another and with malnutrition risk, FCR, and sleep disturbance at W1. Thus, all autoregressive and cross-lagged paths were estimated after adjustment for demographic, anthropometric, endocrine, and surgical factors. The covariate-adjusted model was used as the primary structural model. Participants with incomplete data at any of the four assessment waves were excluded from the primary complete-case analysis. To evaluate potential attrition bias, baseline demographic, clinical, and study variables were compared between participants who completed all four assessments and those who did not. Continuous variables were compared using independent-samples *t*-tests or Mann–Whitney U tests, as appropriate, and categorical variables were compared using chi-square tests or Fisher’s exact tests.

A cross-lagged panel model (CLPM) was constructed using AMOS 24.0 to examine the longitudinal reciprocal relationships among malnutrition risk, FCR, and sleep disturbance (24). The model simultaneously estimated (1) autoregressive paths to assess the temporal stability of each variable across adjacent time points (W1→W2, W2→W3, W3→W4), (2) cross-lagged paths to evaluate prospective temporal associations between variables across adjacent waves, and (3) synchronous correlations among residuals within the same time point. Model fit was evaluated using multiple indices, including the chi-square statistic (χ^2^), the ratio of chi-square to degrees of freedom (χ^2^/df), the comparative fit index (CFI), the Tucker-Lewis index (TLI), the goodness-of-fit index (GFI), and the root mean square error of approximation (RMSEA). Acceptable model fit was indicated by χ^2^/df < 5, CFI and TLI > 0.90, GFI > 0.90, and RMSEA < 0.08. To address the limitation that the conventional CLPM may conflate stable between-person differences with within-person temporal fluctuations, a random-intercept cross-lagged panel model (RI-CLPM) was additionally conducted as a sensitivity analysis. The RI-CLPM included a random intercept for each construct to capture time-invariant between-person differences. The autoregressive and cross-lagged paths were then estimated among the wave-specific within-person deviation components. Accordingly, the RI-CLPM cross-lagged coefficients represent whether a temporary deviation from an individual’s expected level in one construct was prospectively associated with a subsequent deviation in another construct. The same covariates used in the primary adjusted CLPM were included in the RI-CLPM. Model fit was evaluated using χ^2^/df, CFI, TLI, and RMSEA. The RI-CLPM was used only as a sensitivity analysis to assess whether the principal CLPM findings were robust after separating stable between-person differences from within-person fluctuations. Neither model was interpreted as establishing causal relationships.

## 3 Results

### 3.1 Descriptive statistics and correlations

Pearson correlations for the major study variables across the four waves are presented in Table 1. A total of 940 patients were enrolled at W1. Of these, 922 participants completed the W2 assessment, 907 completed the W3 assessment, and 893 completed the W4 assessment, corresponding to retention rates of 98.1%, 96.5%, and 95.0%, respectively. Overall, 47 participants did not complete all four assessments. The primary longitudinal analyses therefore included 893 complete cases. Participants who completed all four assessments did not differ significantly from those who did not with respect to baseline age, sex, BMI, marital status, educational level, malnutrition risk, FCR, sleep disturbance, surgical extent, or postoperative thyroid-function indicators (all *p* > 0.05). In summary, the mean age of the participants was 42.01 years (standard deviation = 14.2). There were more females (54.1%) than males (45.9%), the majority of participants were married (75.4%), a significant proportion were still working (65.2%), and 53.1% had an education level below high school. The average BMI was 23.15 ± 4.12. The median postoperative TSH level was 0.18 mIU/L (IQR, 0.08–0.42), whereas the mean FT4 and FT3 levels were 18.6 ± 3.4 pmol/L and 5.1 ± 0.9 pmol/L, respectively. A total of 273 patients (30.6%) underwent lobectomy, and 620 patients (69.4%) underwent total thyroidectomy. The malnutrition risk score showed a slow upward trend over the follow-up period: from 1.26 at W1 to 2.30 at W4. In contrast, FCR and sleep disturbance gradually declined over the follow-up period. Specifically, the mean FCR score decreased from 9.20 at W1 to 7.55 at W4, while the mean sleep disturbance score declined from 11.76 to 9.53. As shown in Table 1, all study variables were significantly and positively correlated both within and across waves. Within-wave correlations indicated that malnutrition risk, FCR, and sleep disturbance were moderately associated with one another at each time point. Significant correlations across adjacent and non-adjacent waves were observed for all three constructs, providing preliminary evidence of temporal continuity over the 6-month postoperative period (Table 1). These findings provided preliminary support for subsequent longitudinal structural analyses and highlighted the persistence of symptom burden during postoperative recovery, a period that may be particularly relevant to integrated supportive care.

**TABLE 1:** Pearson correlations among study variables across four waves (*N* = 893).

Variables	M	SD	1	2	3	4	5	6	7	8	9	10	11	12
Malnutrition risk (W1)	1.26	0.81	–											
FCR (W1)	9.20	2.80	0.40^***^	–										
Sleep disturbance (W1)	11.76	1.82	0.35^***^	0.47^***^	–									
Malnutrition risk (W2)	1.76	0.82	0.48^***^	0.32^***^	0.23^***^	–								
FCR (W2)	8.45	2.68	0.33^***^	0.40^***^	0.28^***^	0.55^***^	–							
Sleep disturbance (W2)	10.98	3.13	0.22^***^	0.46^***^	0.43^***^	0.48^***^	0.67^***^	–						
Malnutrition risk (W3)	2.25	0.68	0.27^***^	0.31^***^	0.40^***^	0.28^***^	0.57^**^	0.25^***^	–					
FCR (W3)	7.90	2.60	0.17^***^	0.34^***^	0.47^***^	0.26^***^	0.48^***^	0.32^***^	0.31^***^	–				
Sleep disturbance (W3)	9.96	1.91	0.32^***^	0.41^***^	0.39^***^	0.26^***^	0.37^***^	0.38^***^	0.50^***^	0.55^***^	–			
Malnutrition risk (W4)	2.30	0.71	0.58^***^	0.46^***^	0.42^***^	0.30^***^	0.26^***^	0.21^***^	0.26^***^	0.46^***^	0.40^***^	–		
FCR (W4)	7.55	2.55	0.59^***^	0.51^***^	0.51^***^	0.45^***^	0.29^***^	0.37^***^	0.46^**^	0.36^***^	0.22^***^	0.34^***^	–	
Sleep disturbance (W4)	9.53	2.05	0.16^***^	0.50^***^	0.37^***^	0.29^***^	0.17^***^	0.37^***^	0.38^***^	0.12^**^	0.61^***^	0.42^***^	0.51^***^	–

*p < 0.05, ^**^p < 0.01, ^***^p < 0.001; W1: 3-days post-surgery, W2: 1-month post-surgery, W3: 3-month post-surgery, W4: 6-month post-surgery. FCR: fear of cancer recurrence.

### 3.2 Measurement invariance across time

Longitudinal measurement invariance was tested prior to structural modeling, and the results are presented in Table 2. For malnutrition risk, configural, metric, and scalar invariance were all supported across the four waves. Although the configural model for malnutrition risk showed a slightly marginal CFI, the changes in fit indices across increasingly constrained models remained within recommended thresholds. Specifically, for all three constructs, the changes in CFI and RMSEA were below the recommended cutoffs of 0.01 and 0.015, respectively, indicating acceptable measurement invariance over time. These results suggest that malnutrition risk, FCR, and sleep disturbance were measured consistently across the four waves, thereby supporting the comparability of the latent constructs and justifying the use of cross-lagged panel modeling.

**TABLE 2:** Measurement invariance testing across four waves.

Variables	χ ^2^	*df*	CFI	Δ CFI	TLI	Δ TLI	RMSEA	Δ RMSEA
Malnutrition risk								
Configural invariance	849.257	359	0.894		0.894		0.049	
Metric invariance	863.573	373	0.894	0.000	0.898	0.004	0.047	0.002
Scalar invariance	888.794	389	0.893	0.001	0.901	0.003	0.045	0.002
Fear of cancer recurrence								
Configural invariance	216.784	103	0.890		0.857		0.046	
Metric invariance	222.696	113	0.896	0.006	0.877	0.020	0.042	0.004
Scalar invariance	228.700	127	0.903	0.007	0.896	0.019	0.039	0.003
Sleep disturbance								
Configural invariance	222.867	103	0.902		0.887		0.047	
Metric invariance	228.793	128	0.905	0.003	0.901	0.014	0.049	0.002
Scalar invariance	234.395	142	0.914	0.009	0.917	0.016	0.046	0.003

ΔCFI < 0.01 and ΔRMSEA < 0.015 indicate acceptable invariance.

### 3.3 Cross-lagged panel model

**FIGURE 1:**
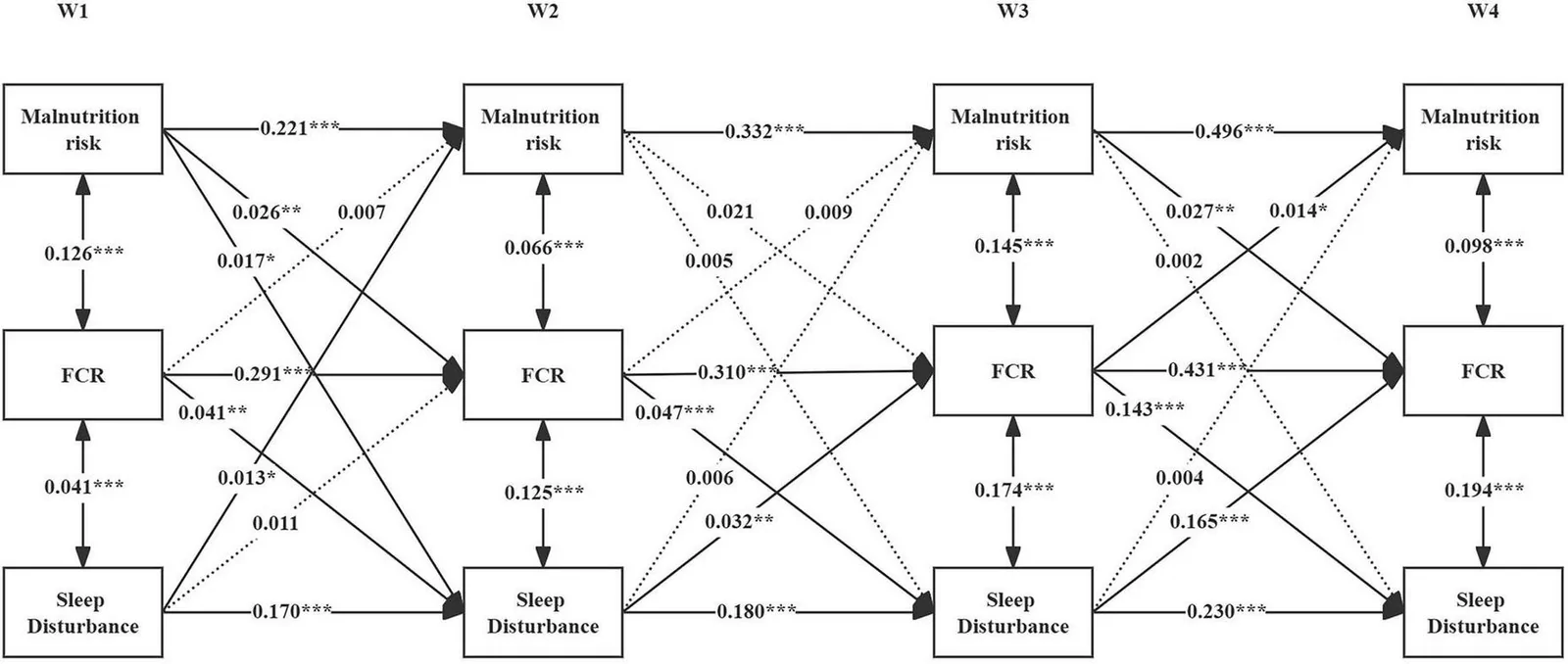
A cross-lagged model of the longitudinal relationships among malnutrition risk, fear of cancer recurrence, and sleep disturbance in PTC. The single arrow represents the regression path and the double arrow represents the correlation. All the reported parameters are standardized. W1, W2, W3, W4 = Wave 1, 2, 3, 4. *p < 0.05; **p < 0.01; ***p < 0.001. FCR, fear of cancer recurrence.

The covariate-adjusted cross-lagged panel model demonstrated acceptable model fit: χ^2^ (25) = 44.315, *p* = 0.019, χ^2^/df = 1.641, CFI = 0.981, TLI = 0.925, RMSEA = 0.027, and 90% CI [0.011, 0.041]. Among the covariates, higher baseline BMI was significantly associated with malnutrition risk at W2 and W3, whereas female sex was significantly associated with higher FCR at W2. The remaining covariate paths were not statistically significant. After adjustment for age, sex, BMI, marital status, educational level, postoperative TSH, FT4, FT3, and surgical extent, the direction, magnitude, and statistical significance of the principal autoregressive and cross-lagged associations were largely unchanged. The adjusted estimates were comparable with those obtained from the unadjusted model. Accordingly, the covariate-adjusted model is presented as the primary model in Figure 1.

The RI-CLPM demonstrated good model fit: χ^2^ (58) = 76.43, *p* = 0.053, CFI = 0.991, TLI = 0.985, RMSEA = 0.019, and 90% CI [0.007, 0.031]. After separating stable between-person differences from within-person fluctuations, all three constructs showed significant within-person autoregressive associations across adjacent waves. Regarding the cross-lagged associations, higher-than-usual FCR at W1, W2, and W3 was prospectively associated with higher-than-usual sleep disturbance at W2, W3, and W4, respectively (β = 0.033, 0.041, and 0.038, respectively; all *p* < 0.001). Higher-than-usual sleep disturbance at W2 and W3 was prospectively associated with higher-than-usual FCR at W3 and W4, respectively (β = 0.028, *p* < 0.01; β = 0.034, *p* < 0.001). Higher-than-usual malnutrition risk at W1 was prospectively associated with higher-than-usual FCR at W2 (β = 0.019, *p* = 0.014), and higher-than-usual FCR at W3 was prospectively associated with higher-than-usual malnutrition risk at W4 (β = 0.012, *p* = 0.031). In contrast, the cross-lagged associations from malnutrition risk at W1 to sleep disturbance at W2 (β = 0.009, *p* = 0.093) and from malnutrition risk at W3 to FCR at W4 (β = 0.011, *p* = 0.078) were not statistically significant. Overall, the RI-CLPM supported a consistent prospective within-person association from FCR to subsequent sleep disturbance and a reciprocal pattern between FCR and sleep disturbance during the later recovery stages. Some of the weaker associations involving malnutrition risk were attenuated or not replicated after stable between-person differences were separated. The model structure and standardized path estimates are shown in Supplementary Table 2.

#### 3.3.1 Autoregressive effects

All three core variables demonstrated significant autoregressive effects across adjacent waves, indicating temporal stability over time. Malnutrition risk showed significant stability from W1 to W2 (β = 0.221, *p* < 0.001), from W2 to W3 (β = 0.332, *p* < 0.001), and from W3 to W4 (β = 0.496, *p* < 0.001). Similarly, FCR exhibited significant autoregressive effects from W1 to W2 (β = 0.291, *p* < 0.001), from W2 to W3 (β = 0.310, *p* < 0.001), and from W3 to W4 (β = 0.431, *p* < 0.001). Sleep disturbance also showed significant but comparatively smaller autoregressive effects across the three intervals (W1→W2: β = 0.170, *p* < 0.001; W2→W3: β = 0.180, *p* < 0.001; W3→W4: β = 0.230, *p* < 0.001).

#### 3.3.2 Cross-lagged effects from W1 to W2

During the early postoperative stage, malnutrition risk at W1 was prospectively associated with FCR at W2 (β = 0.026, *p* < 0.01) and sleep disturbance at W2 (β = 0.017, *p* < 0.05). FCR at W1 was prospectively associated with sleep disturbance at W2 (β = 0.041, *p* < 0.01), but the cross-lagged association from FCR at W1 to malnutrition risk at W2 was not statistically significant (β = 0.007, *p* > 0.05). In addition, sleep disturbance at W1 was prospectively associated with malnutrition risk at W2 (β = 0.013, *p* < 0.05), whereas the corresponding cross-lagged association with FCR was not statistically significant (β = 0.011, *p* > 0.05).

#### 3.3.3 Cross-lagged effects from W3 to W4

From W3 to W4, malnutrition risk was prospectively associated with subsequent FCR (β = 0.027, *p* < 0.01), but not subsequent sleep disturbance (β = 0.002, *p* > 0.05). FCR was prospectively associated with both subsequent malnutrition risk (β = 0.014, *p* < 0.05) and subsequent sleep disturbance (β = 0.143, *p* < 0.001). In addition, sleep disturbance was prospectively associated with subsequent FCR (β = 0.165, *p* < 0.001), whereas its effect on malnutrition risk was not significant (β = 0.004, *p* > 0.05).

#### 3.3.4 Within-wave associations

Significant within-wave associations were observed among the latent variables at each assessment wave. Malnutrition risk was positively associated with FCR at W1 (β = 0.126, *p* < 0.001), W2 (β = 0.066, *p* < 0.001), W3 (β = 0.145, *p* < 0.001), and W4 (β = 0.098, *p* < 0.001). Similarly, FCR was positively associated with sleep disturbance at W1 (β = 0.041, *p* < 0.001), W2 (β = 0.125, *p* < 0.001), W3 (β = 0.174, *p* < 0.001), and W4 (β = 0.194, *p* < 0.001). These results indicate that the three constructs were not only longitudinally linked across time but also concurrently correlated within each postoperative stage.

### 3.4 Summary of longitudinal relationships

Overall, the cross-lagged findings revealed several distinct longitudinal patterns. First, FCR showed consistent prospective associations with subsequent sleep disturbance across all three intervals, suggesting that elevated FCR may represent an earlier correlate of poorer subsequent sleep during postoperative recovery. Second, the cross-lagged association from sleep disturbance to subsequent FCR emerged during the later follow-up periods, becoming significant from W2 to W3 and from W3 to W4, indicating a progressively reciprocal longitudinal relationship between these two variables. Third, the longitudinal association between malnutrition risk and FCR appeared to vary by recovery stage: malnutrition risk was prospectively associated with subsequent FCR at W1→W2 and W3→W4, whereas FCR was prospectively associated with subsequent malnutrition risk only at W3→W4. Finally, reciprocal cross-lagged associations between malnutrition risk and sleep disturbance were observed only during the early postoperative stage. Taken together, these findings suggest that the temporal associations among malnutrition risk, FCR, and sleep disturbance are stage-dependent and become increasingly differentiated over the course of recovery. The RI-CLPM sensitivity analysis broadly supported the principal FCR–sleep disturbance findings, although several weaker associations involving malnutrition risk were attenuated. Although several cross-lagged associations reached statistical significance, the standardized coefficients were small. Such modest associations are common in longitudinal studies of complex, multifactorial behavioral and psychological outcomes and should not be interpreted as indicating strong individual-level effects.

## 4 Discussion

The present study examined the longitudinal reciprocal relationships among malnutrition risk, sleep disturbance, and fear of cancer recurrence in patients with PTC from the immediate postoperative period to 6 months after surgery. Malnutrition risk gradually increased over follow-up, whereas FCR and sleep disturbance declined. All three constructs showed significant temporal stability, although sleep disturbance was less stable than malnutrition risk and FCR. The cross-lagged associations also differed by recovery stage. FCR showed consistent prospective associations with subsequent sleep disturbance across all intervals, while the prospective association from sleep disturbance to subsequent FCR emerged only during later recovery. In contrast, the association between malnutrition risk and FCR varied by stage, and reciprocal cross-lagged associations between malnutrition risk and sleep disturbance were limited to the early postoperative phase. Overall, postoperative recovery in PTC appears to involve a dynamic interplay among nutritional vulnerability, sleep, and recurrence-related worry rather than a simple linear improvement. The RI-CLPM sensitivity analysis provided additional information by separating stable between-person differences from within-person fluctuations. The prospective association from FCR to subsequent sleep disturbance was consistent across all three intervals in both the CLPM and RI-CLPM. In addition, the reciprocal pattern between FCR and sleep disturbance during the later recovery stages (W2→W3 and W3→W4) was also replicated. These convergent findings suggest that the observed associations were not attributable solely to stable between-person differences. Nevertheless, several associations identified in the conventional CLPM were attenuated in the RI-CLPM, indicating that they may have partly reflected stable between-person differences rather than within-person temporal processes. Specifically, the cross-lagged paths from malnutrition risk at W1 to sleep disturbance at W2, from sleep disturbance at W1 to malnutrition risk at W2, and from malnutrition risk at W3 to FCR at W4, all of which were statistically significant in the CLPM, did not reach statistical significance in the RI-CLPM. The CLPM and RI-CLPM findings should therefore be interpreted as complementary rather than interchangeable. The RI-CLPM sensitivity analysis broadly supported the main pattern involving FCR and sleep disturbance. However, several smaller cross-lagged associations involving malnutrition risk were attenuated, suggesting that these weaker findings should be interpreted cautiously.

### 4.1 Longitudinal trajectories of malnutrition risk, fear of cancer recurrence, and sleep disturbance

The three symptom domains followed different trajectories over time. FCR and sleep disturbance gradually decreased, whereas malnutrition risk increased. This suggests that recovery after PTC surgery is not uniformly favorable (25). Early after surgery, patients may experience greater recurrence-related worry and transient sleep disruption because of uncertainty regarding pathology, treatment decisions, and bodily changes (26). As recovery progresses, these acute concerns may ease. By contrast, nutritional vulnerability may become more prominent later, when cumulative treatment burden, dietary disruption, endocrine adjustment, reduced appetite, and fatigue-related self-management difficulties increasingly affect daily life (27). The upward trend in malnutrition risk suggests that nutritional problems may emerge gradually rather than being limited to the immediate postsurgical phase. The increase in PG-SGA scores should not be interpreted exclusively as progressive malnutrition. It may partly reflect endocrine-related symptoms, postoperative functional changes, treatment adjustment, or other non-nutritional contributors. Recovery after PTC often extends beyond wound healing and involves longer-term adjustment to hormonal therapy, symptom monitoring, and lifestyle change (28). Nutritional risk during this period may therefore reflect not only physical symptoms but also broader behavioral and psychosocial processes. These results support repeated, stage-sensitive nutritional screening rather than assuming that favorable oncologic outcomes necessarily indicate optimal nutritional recovery (29).

### 4.2 Prospective association between fear of cancer recurrence and subsequent sleep disturbance

The most consistent longitudinal pattern was the prospective association from FCR to subsequent sleep disturbance across all follow-up intervals. Recurrence-related worry showed a consistent but small prospective association with subsequent sleep disturbance and may therefore warrant consideration during clinical assessment (30). Patients with elevated FCR are likely to engage in persistent body vigilance, repeated symptom monitoring, and worry about disease progression, all of which can interfere with sleep initiation and maintenance (31). This pattern suggests that sleep disturbance in this population may reflect not only surgery-related or endocrine factors but also sustained cognitive-emotional activation, but may also reflect sustained cognitive-emotional activation. As a result, sleep-focused interventions may be less effective if they address behavioral sleep hygiene alone and overlook recurrence-related cognitions (32). Persistent sleep complaints after thyroid cancer surgery may therefore warrant routine assessment of FCR rather than being treated as isolated symptoms.

### 4.3 Delayed reciprocal associations between sleep disturbance and fear of cancer recurrence

Sleep disturbance was prospectively associated with subsequent FCR only during the later follow-up periods, indicating that the relationship between these two constructs becomes increasingly reciprocal over time (33). Early in recovery, elevated FCR preceded greater subsequent sleep disturbance. During later recovery, however, persistent sleep disruption may be associated with greater subsequent recurrence-related concern. Ongoing sleep problems may heighten fatigue, emotional reactivity, and sensitivity to bodily discomfort, making patients more likely to interpret persistent symptoms as signs of incomplete recovery or recurrence (34–36). In this way, sleep disturbance may gradually become part of the patient’s illness interpretation process (31). Clinically, this suggests different priorities at different stages: reducing FCR may help prevent sleep deterioration early after surgery, whereas improving sleep may become increasingly relevant to lowering recurrence-related fear later in recovery.

### 4.4 Stage-specific interplay between malnutrition risk and fear of cancer recurrence

The relationship between malnutrition risk and FCR was more variable. Malnutrition risk was prospectively associated with subsequent FCR during the early and later stages, whereas FCR was prospectively associated with subsequent malnutrition risk during the later stage. This indicates that the link between nutritional vulnerability and recurrence-related worry changes over time. In the early stage, higher malnutrition risk may reflect a more difficult recovery experience and increase concern about disease progression or incomplete healing. Eating difficulties, weight change, fatigue, and reduced physical functioning may be interpreted as warning signs, potentially accompanying greater subsequent FCR. In the later stage, the reverse path became significant, suggesting that persistent recurrence-related worry may be associated with poorer subsequent self-management and daily regulation (37). Reduced appetite, disrupted routines, and poorer adherence to recovery-related behaviors may then contribute to worsening nutritional vulnerability. This shifting pattern is particularly relevant for nutrition-oriented survivorship care. Nutritional screening during follow-up should not be separated from psychological assessment, because nutritional vulnerability and recurrence-related fear may show stage-dependent reciprocal associations.

### 4.5 Early reciprocal associations between malnutrition risk and sleep disturbance

Reciprocal cross-lagged associations between malnutrition risk and sleep disturbance were observed during the early postoperative phase in the conventional CLPM, suggesting that this period may represent a clinically reactive window in which nutritional vulnerability and sleep disturbance are closely interrelated (38, 39). Early sleep problems may worsen fatigue, appetite regulation, and daytime functioning, thereby increasing nutritional risk. Conversely, early nutritional vulnerability may reflect pain, reduced intake, gastrointestinal discomfort, weakness, or general physical stress, all of which may impair sleep. When acute surgical stress subsides, this relationship becomes less direct and may be increasingly shaped by FCR and broader survivorship processes. From a symptom network perspective, the dominant bridge connecting nutritional and behavioral symptoms may therefore change over time (40, 41). Early after surgery, malnutrition risk and sleep disturbance appear closely linked, whereas later recovery may be more strongly organized around FCR. However, these relatively small associations were not replicated in the RI-CLPM sensitivity analysis and should therefore be interpreted cautiously.

### 4.6 Theoretical and clinical implications

This study extends existing thyroid cancer survivorship research by showing that malnutrition risk, sleep disturbance, and FCR are not merely co-occurring problems, but dynamically interrelated longitudinal processes that change across postoperative recovery. Early after surgery, nutritional vulnerability and sleep disturbance are more tightly coupled, whereas later recovery is increasingly characterized by reciprocal prospective associations involving FCR. Clinically, these findings support a stage-matched survivorship care model. Follow-up after PTC surgery should include repeated assessment of nutritional risk, sleep disturbance, and FCR rather than focusing only on recurrence surveillance and general symptom burden. In the immediate postoperative phase, nutritional vulnerability and sleep problems may require particular attention. Later recovery may require greater focus on recurrence-related worry and its subsequent associations with sleep and nutritional self-management. Although several cross-lagged associations were statistically significant, their standardized coefficients were small. Given the large sample size, statistical significance does not necessarily imply substantial clinical importance. The findings therefore indicate modest longitudinal interrelationships rather than strong predictive effects. For example, the association between FCR and subsequent sleep disturbance may identify a clinically relevant pattern warranting attention, but the relative contribution of FCR to later sleep disturbance appears limited. These results should not be interpreted as evidence that changes in one domain would necessarily produce clinically meaningful changes in another.

### 4.7 Limitations

Several limitations should be noted. First, the use of a single-center convenience sample from one geographic region may have introduced selection bias and limited the representativeness and external validity of the findings. Treatment practices, patient demographic characteristics, healthcare access, and cultural factors may differ across institutions and regions, potentially influencing the observed longitudinal associations. Future multicenter studies involving more diverse populations are needed to validate the present findings. Second, all variables were assessed using self-report instruments or clinically scored scales, which may still be influenced by reporting bias or measurement context. Although postoperative TSH, FT4, and FT3 levels were included as covariates, residual confounding related to fluctuations in thyroid function and treatment adjustment cannot be completely excluded. Although the exclusion of patients with current or previous psychiatric disorders or recent psychotropic medication use was intended to reduce confounding, it represents an important source of selection bias. This criterion may have resulted in a healthier and less symptomatic sample, potentially underestimating the levels and severity of FCR and sleep disturbance and limiting the generalizability of the findings to patients with comorbid psychiatric conditions. Although the RI-CLPM sensitivity analysis separated stable between-person differences from within-person fluctuations, it does not establish causality and remains dependent on model assumptions and the absence of unmeasured time-varying confounding. Therefore, all cross-lagged findings should be interpreted as prospective associations rather than causal effects. The 6-month follow-up period was relatively short for evaluating long-term nutritional status. Although the PG-SGA provides a multidimensional assessment of malnutrition risk and is sensitive to changes in food intake, nutrition-impact symptoms, and functional status, the observed changes cannot be attributed exclusively to malnutrition. Postoperative reductions in physical activity, endocrine and medication adjustment, treatment-related symptoms, age-related factors, and other iatrogenic or behavioral influences may also have contributed. Future studies with longer follow-up and repeated objective assessments of body composition, muscle strength, dietary intake, and physical activity are needed to characterize long-term nutritional trajectories more accurately. The observed cross-lagged effect sizes were small. Although several associations reached statistical significance, their clinical importance should be interpreted cautiously, particularly given the large sample size. These findings should be regarded as hypothesis-generating signals that warrant further investigation using clinically anchored outcomes and intervention-based designs. Future studies should examine these longitudinal relationships in more inclusive, real-world clinical populations, including patients with psychiatric comorbidities and those receiving psychotropic treatment.

## 5 Conclusion

In conclusion, malnutrition risk, FCR, and sleep disturbance showed dynamic and stage-dependent prospective associations during postoperative recovery in patients with PTC. FCR was consistently associated with subsequent sleep disturbance across all follow-up intervals, whereas reciprocal prospective associations between sleep disturbance and FCR emerged during later recovery. The association between malnutrition risk and FCR varied by recovery stage, and reciprocal cross-lagged associations between malnutrition risk and sleep disturbance were observed during the early postoperative period. These findings should be interpreted as prospective longitudinal associations rather than causal effects. Repeated screening and integrated supportive care, including nutritional monitoring, sleep management, and FCR assessment, may therefore be relevant to postoperative recovery in this population.

## Data Availability

The raw data supporting the conclusions of this article will be made available by the authors, without undue reservation.

## References

[1] Caron NR Clark OH. Papillary thyroid cancer. *Curr Treat Options Oncol*. (2006) 7:309–19. 10.1007/s11864-006-0040-7 16916491

[2] Haymart P Levin NJ Haymart MR. The psychosocial impact of thyroid cancer. *Curr Opin Endocrinol Diabetes Obes*. (2023) 30:252–8. 10.1097/MED.0000000000000815 37288721 PMC10526714

[3] Aschebrook-Kilfoy B James B Nagar S Kaplan S Seng V Ahsan H et al. Risk factors for decreased quality of life in thyroid cancer survivors: initial findings from the North American thyroid cancer survivorship study. *Thyroid.* (2015) 25:1313–21. 10.1089/thy.2015.0098 26431811 PMC4684649

[4] Hampton J Alam A Zdenkowski N Rowe C Fradgley E O’Neill CJ. Fear of cancer recurrence in differentiated thyroid cancer survivors: a systematic review. *Thyroid.* (2024) 34:541–58. 10.1089/thy.2023.0642 38368547

[5] Koo DL Park Y Nam H Chai YJ. Sleep quality of patients with papillary thyroid carcinoma: a prospective longitudinal study with 5-year follow-up. *Sci Rep*. (2022) 12:18823. 10.1038/s41598-022-23549-3 36335214 PMC9637156

[6] Jager-Wittenaar H Ottery FD. Assessing nutritional status in cancer: role of the patient-generated subjective global assessment. *Curr Opin Clin Nutr Metab Care*. (2017) 20:322–9. 10.1097/MCO.0000000000000389 28562490

[7] Lang J Shao Y Liao J Chen J Zhou X Deng R et al. Patient-Generated Subjective Global Assessment (PG-SGA) predicts length of hospital stay in lung adenocarcinoma patients. *Br J Nutr*. (2022) 127:1543–8. 10.1017/S0007114521003500 34503589 PMC9044215

[8] Plyta M Patel PS Fragkos KC Kumagai T Mehta S Rahman F et al. Nutritional status and quality of life in hospitalised cancer patients who develop intestinal failure and require parenteral nutrition: an observational study. *Nutrients*. (2020) 12:2357. 10.3390/nu12082357 32784602 PMC7468734

[9] Kotwal A Fingeret A Knape A Patel A Bradford Bell E Goldner W. Thyroid cancer survivorship: challenges and opportunities. *Endocr Pract*. (2024) 30:1097–102. 10.1016/j.eprac.2024.08.003 39209023

[10] Agate L Minaldi E Basolo A Angeli V Jaccheri R Santini F et al. Nutrition in advanced thyroid cancer patients. *Nutrients*. (2022) 14:1298. 10.3390/nu14061298 35334955 PMC8951395

[11] He Y Meng Z Jia Q Hu F He X Tan J et al. Sleep quality of patients with differentiated thyroid cancer. *PLoS One*. (2015) 10:e0130634. 10.1371/journal.pone.0130634 26083787 PMC4471198

[12] Ioannou E Ruhrberg C. Whole-mount immunofluorescence protocol for 3d imaging, reconstruction, and quantification of fourth pharyngeal arch artery formation. *Methods Mol Biol*. (2022) 2441:41–62. 10.1007/978-1-0716-2059-5_4 35099727

[13] Rimmer B Brown MC Sotire T Beyer F Bolnykh I Balla M et al. Characteristics and components of self-management interventions for improving quality of life in cancer survivors: a systematic review. *Cancers*. (2023) 16:14. 10.3390/cancers16010014 38201442 PMC10777971

[14] Maki Y Horiuchi K Okamoto T. Fatigue and quality of life among thyroid cancer survivors without persistent or recurrent disease. *Endocr Connect*. (2022) 11:e210506. 10.1530/EC-21-0506 35107083 PMC8942325

[15] Rogers SN Mepani V Jackson S Lowe D. Health-related quality of life, fear of recurrence, and emotional distress in patients treated for thyroid cancer. *Br J Oral Maxillofac Surg*. (2017) 55:666–73. 10.1016/j.bjoms.2016.09.001 28648407

[16] Tagay S Herpertz S Langkafel M Erim Y Bockisch A Senf W et al. Health-related Quality of Life, depression and anxiety in thyroid cancer patients. *Qual Life Res*. (2006) 15:695–703. 10.1007/s11136-005-3689-7 16688502

[17] Ottery FD. Rethinking nutritional support of the cancer patient: the new field of nutritional oncology. *Semin Oncol.* (1994) 21:770–8. 7992092

[18] Bauer J Capra S Ferguson M. Use of the scored Patient-Generated Subjective Global Assessment (PG-SGA) as a nutrition assessment tool in patients with cancer. *Eur J Clin Nutr*. (2002) 56:779–85. 10.1038/sj.ejcn.1601412 12122555

[19] Humphris GM Watson E Sharpe M Ozakinci G. Unidimensional scales for fears of cancer recurrence and their psychometric properties: the FCR4 and FCR7. *Health Qual Life Outcomes*. (2018) 16:30. 10.1186/s12955-018-0850-x 29471823 PMC5822647

[20] Buysse DJ Reynolds CF Monk TH Berman SR Kupfer DJ. The Pittsburgh Sleep Quality Index: a new instrument for psychiatric practice and research. *Psychiatry Res*. (1989) 28:193–213. 10.1016/0165-1781(89)90047-4 2748771

[21] Guo X Qin Z Meng C Lv J Hu Y Fei J et al. Investigation of the sleep quality among hospitalized cardiovascular patients using regression models and qualitative comparative analysis. *Clin Nurs Res*. (2023) 32:580–8. 10.1177/10547738221148150 36633212

[22] Meredith W. Measurement invariance, factor-analysis and factorial invariance. *Psychometrika.* (1993) 58:525–43. 10.1007/Bf02294825

[23] Chen FF. Sensitivity of goodness of fit indexes to lack of measurement invariance. *Struct Equ Model.* (2007) 14:464–504. 10.1080/10705510701301834

[24] Kenny DA Harackiewicz JM. Cross-lagged panel correlation: practice and promise. *J Appl Psychol.* (1979) 64:372–9. 10.1037/0021-9010.64.4.372

[25] Borson-Chazot F Terra JL Goichot B Caron P. What is the quality of life in patients treated with levothyroxine for hypothyroidism and how are we measuring it? A critical, narrative review. *J Clin Med*. (2021) 10:1386. 10.3390/jcm10071386 33808358 PMC8037475

[26] Chang DJ Leung AM. Who’s going to manage the thyroid cancer? *J Clin Endocrinol Metab*. (2020) 105:e3820–1. 10.1210/clinem/dgaa514 32777046 PMC7456339

[27] Applewhite MK James BC Kaplan SP Angelos P Kaplan EL Grogan RH et al. Quality of life in thyroid cancer is similar to that of other cancers with worse survival. *World J Surg*. (2016) 40:551–61. 10.1007/s00268-015-3300-5 26546191

[28] Berrett-Abebe J Cadet T Pirl W Lennes I. Exploring the relationship between fear of cancer recurrence and sleep quality in cancer survivors. *J Psychosoc Oncol*. (2015) 33:297–309. 10.1080/07347332.2015.1020586 25751193

[29] Arditte Hall KA Price SN Lucas AR Park ER Wagner LI Mizrach HR et al. An exploration into the relationship between insomnia and repetitive negative thinking among cancer survivors. *J Psychosoc Oncol*. (2025) 43:59–72. 10.1080/07347332.2024.2356193 38831557 PMC11612031

[30] Li N Wensley C Reynolds L Baraza W Leyton TO Jull A. Interventions to reduce fear of cancer recurrence among people with cancer: scoping review. *JMIR Cancer*. (2026) 12:e81579. 10.2196/81579 42018993 PMC13102330

[31] Perndorfer C Soriano EC Siegel SD Spencer RMC Otto AK Laurenceau JP. Fear of cancer recurrence and sleep in couples coping with early-stage breast cancer. *Ann Behav Med*. (2022) 56:1131–43. 10.1093/abm/kaac018 35551585 PMC9635995

[32] Yarosh RA Jackson CL Anderson C Nichols HB Sandler DP. Sleep disturbances among cancer survivors. *Cancer Epidemiol*. (2023) 87:102471. 10.1016/j.canep.2023.102471 37837808 PMC10873004

[33] Strollo SE Fallon EA Gapstur SM Smith TG. Cancer-related problems, sleep quality, and sleep disturbance among long-term cancer survivors at 9-years post diagnosis. *Sleep Med*. (2020) 65:177–85. 10.1016/j.sleep.2019.10.008 32029206

[34] Amani O Mazaheri MA Malekzadeh Moghani M Zarani F Hamidi Choolabi R. Mediating effects of rumination on insomnia in cancer survivors: influences of cancer-related fatigue, fear of recurrence, and psychological distress. *Cancer Med*. (2024) 13:e70189. 10.1002/cam4.70189 39305110 PMC11415615

[35] Park KA Kim S Oh EG Kim H Chang HS Kim SH. Factors affecting the health-promoting behavior of thyroid cancer survivors: comparison by stage of cancer survivorship. *Support Care Cancer*. (2022) 30:3429–39. 10.1007/s00520-022-06799-9 34999951 PMC8857080

[36] Li R Li G Wang Y Bao T Lei Y Tian L et al. Psychological distress and sleep disturbance throughout thyroid nodule screening, diagnosis, and treatment. *J Clin Endocrinol Metab*. (2021) 106:e4221–30. 10.1210/clinem/dgab224 33830242

[37] Pitt SC Haymart MR. Breaking down or waking up? psychological distress and sleep disturbance in patients with thyroid nodules and cancer. *J Clin Endocrinol Metab*. (2021) 106:e4278–80. 10.1210/clinem/dgab319 33982121 PMC8475195

[38] Bergsneider BH Armstrong TS Conley YP Cooper B Hammer M Levine JD et al. Symptom network analysis and unsupervised clustering of oncology patients identifies drivers of symptom burden and patient subgroups with distinct symptom patterns. *Cancer Med*. (2024) 13:e70278. 10.1002/cam4.70278 39377555 PMC11460217

[39] Richard V Gilbert A Pizzolla E Briganti G. Investigating the complexity of multidimensional symptom experiences in patients with cancer: systematic review of the network analysis approach. *JMIR Cancer*. (2025) 11:e66087. 10.2196/66087 40633921 PMC12287671

[40] Browne MW Cudeck R. Alternative ways of assessing model fit. *Sociol Methods Res.* (1992) 21:230–58. 10.1177/0049124192021002005

[41] Bentler PM Bonett DG. Significance tests and goodness of fit in the analysis of covariance structures. *Psychol Bull.* (1980) 88:588–606. 10.1037/0033-2909.88.3.588

