# Supplementary material for: Longitudinal relationships of malnutrition risk, sleep disturbance, and fear of cancer recurrence in thyroid cancer patients: a cross-lagged panel model
Source: Front Nutr. 2026 Aug 21;13:1882241. doi: 10.3389/fnut.2026.1882241 (PMC13541441; doi:10.3389/fnut.2026.1882241)
Supplement: Supplementary file 1 [file Data_Sheet_1.pdf]

Table S1. Coding of covariates included in the adjusted cross-lagged panel model

Covariate	Variable type	Coding
Age	Continuous	Age at enrollment, measured in years
Sex	Binary categorical	0 = Male; 1 = Female
Body mass index (BMI)	Continuous	Body weight in kilograms divided by height in meters squared (kg/m^2)
Marital status	Binary categorical	0 = Unmarried, divorced, or widowed; 1 = Married
Educational level	Binary categorical	0 = Below high school; 1 = High school or above
Postoperative TSH	Continuous	Serum thyroid-stimulating hormone level measured 6 weeks after surgery, in mIU/L
Postoperative FT4	Continuous	Serum free thyroxine level measured 6 weeks after surgery, in pmol/L
Postoperative FT3	Continuous	Serum free triiodothyronine level measured 6 weeks after surgery, in pmol/L
Surgical extent	Binary categorical	0 = Lobectomy; 1 = Total thyroidectomy

Note: *BMI, body mass index; TSH, thyroid-stimulating hormone; FT4, free thyroxine; FT3, free triiodothyronine. Continuous covariates were entered into the model using their observed values. Binary categorical variables were dummy-coded using the categories indicated above.*

Table S2. Standardized Within-Person and Between-Person Parameter Estimates from the Random-Intercept Cross-Lagged Panel Model

Path	β	p
**Autoregressive paths**		
Malnutrition risk W1 → Malnutrition risk W2	0.187	<.001
Malnutrition risk W2 → Malnutrition risk W3	0.268	<.001
Malnutrition risk W3 → Malnutrition risk W4	0.402	<.001
FCR W1 → FCR W2	0.235	<.001
FCR W2 → FCR W3	0.261	<.001
FCR W3 → FCR W4	0.378	<.001
Sleep disturbance W1 → Sleep disturbance W2	0.052	<.001
Sleep disturbance W2 → Sleep disturbance W3	0.143	<.001
Sleep disturbance W3 → Sleep disturbance W4	0.186	<.001
**Cross-lagged paths**		
Malnutrition risk W1 → FCR W2	0.019	.014
Malnutrition risk W1 → Sleep disturbance W2	0.009	.093
FCR W1 → Malnutrition risk W2	0.006	.184
FCR W1 → Sleep disturbance W2	0.033	<.001
Sleep disturbance W1 → Malnutrition risk W2	0.008	.121
Sleep disturbance W1 → FCR W2	0.010	.087
Malnutrition risk W2 → FCR W3	0.008	.142
Malnutrition risk W2 → Sleep disturbance W3	0.007	.198
FCR W2 → Malnutrition risk W3	0.005	.264

Path	β	p
FCR W2 → Sleep disturbance W3	0.041	<.001
Sleep disturbance W2 → Malnutrition risk W3	0.006	.215
Sleep disturbance W2 → FCR W3	0.028	< .01
Malnutrition risk W3 → FCR W4	0.011	.078
Malnutrition risk W3 → Sleep disturbance W4	0.004	.302
FCR W3 → Malnutrition risk W4	0.012	0.031
FCR W3 → Sleep disturbance W4	0.038	<.001
Sleep disturbance W3 → Malnutrition risk W4	0.005	.287
Sleep disturbance W3 → FCR W4	0.034	<.001
**Random intercept correlations**		
Malnutrition risk (RI) ↔ FCR (RI)	0.152	<.001
Malnutrition risk (RI) ↔ Sleep disturbance (RI)	0.089	< .01
FCR (RI) ↔ Sleep disturbance (RI)	0.176	<.001

Note: *FCR = Fear of Cancer Recurrence; RI = Random Intercept; W1–W4 = Waves 1–4. The RI-CLPM also included covariates (age, sex, BMI, marital status, educational level, postoperative TSH, FT4, FT3, and surgical extent), which are not shown here for brevity. All adjacent-wave cross-lagged paths were estimated in the RI-CLPM; non-significant paths are presented for completeness. Autoregressive and cross-lagged coefficients represent within-person associations, whereas random-intercept correlations represent stable between-person associations.*

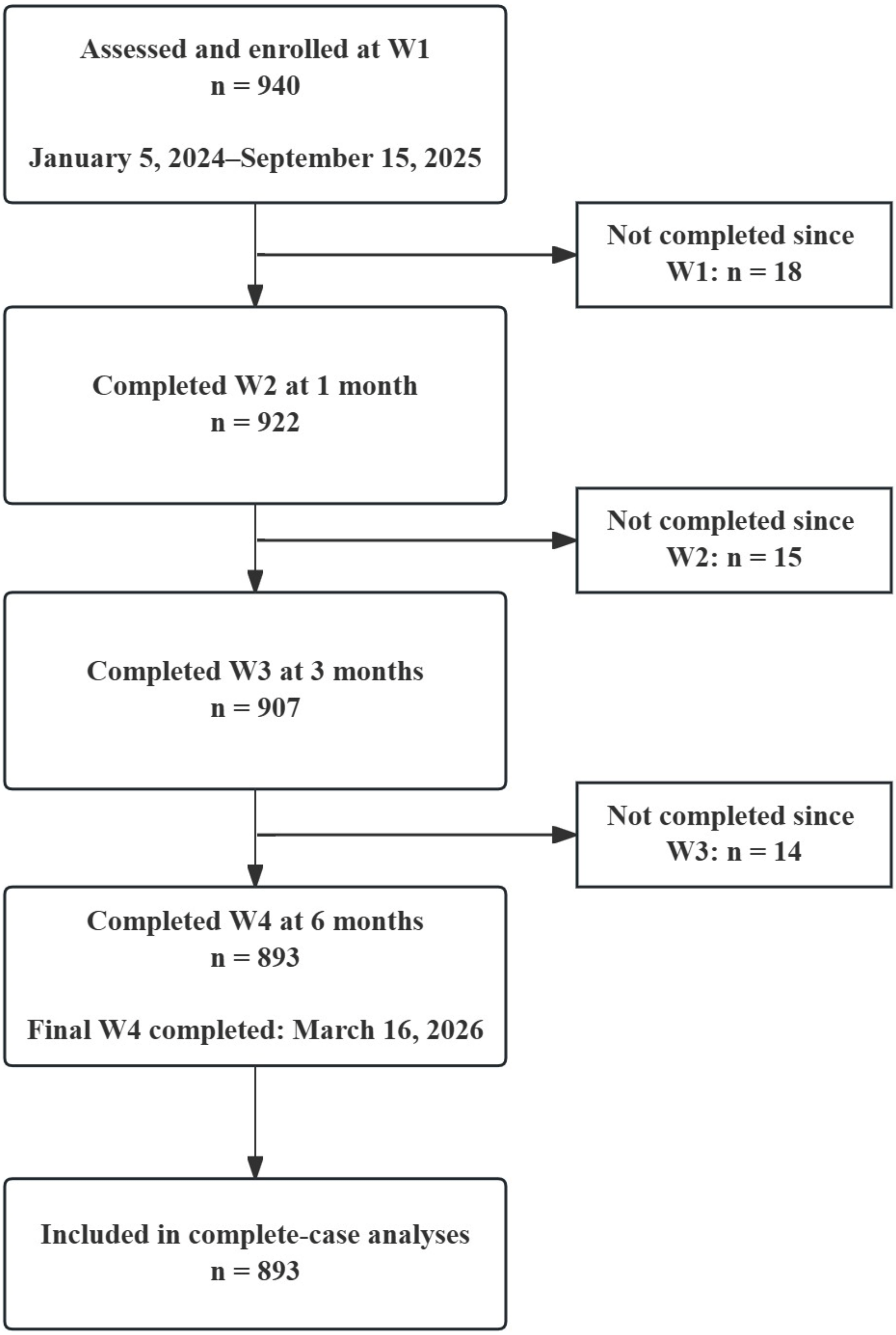

Figure S1 A flowchart of participant selection
